# A novel predictive model based on inflammatory response-related genes for predicting endometrial cancer prognosis and its experimental validation

**DOI:** 10.18632/aging.204767

**Published:** 2023-06-05

**Authors:** Yuting Wang, Bo Wang, Xiaoxin Ma

**Affiliations:** 1 Department of Obstetrics and Gynecology, Shengjing Hospital of China Medical University, Tiexi, Shenyang 110000, Liaoning, People’s Republic of China

**Keywords:** endometrial carcinoma, inflammatory response-related genes (IRGs), prognostic model, immunotherapy response, chemotherapeutic drug sensitivity

## Abstract

Inflammatory response is an important feature of most tumors. Local inflammation promotes tumor cell immune evasion and chemotherapeutic drug resistance. We aimed to build a prognostic model for endometrial cancer patients based on inflammatory response-related genes (IRGs). RNA sequencing and clinical data for uterine corpus endometrial cancer were obtained from TCGA datasets. LASSO-penalized Cox regression was used to obtain the risk formula of the model: the score = e^sum(corresponding coefficient × each gene’s expression)^. The “ESTIMATE” and “pRRophetic” packages in R were used to evaluate the tumor microenvironment and the sensitivity of patients to chemotherapy drugs. Data sets from IMvigor210 were used to evaluate the efficacy of immunotherapy in cancer patients. For experimental verification, 37 endometrial cancer and 43 normal endometrial tissues samples were collected. The mRNA expression of the IRGs was measured using qRT-PCR. The effects of IRGs on the malignant biological behaviors of endometrial cancer were detected using CCK-8, colony formation, Transwell invasion, and apoptosis assays. We developed a novel prognostic signature comprising 13 IRGs, which is an independent prognostic marker for endometrial cancer. A nomogram was developed to predict patient survival accurately. Three key IRGs (LAMP3, MEP1A, and ROS1) were identified in this model. Furthermore, we verified the expression of the three key IRGs using qRT-PCR. Functional experiments also confirmed the influence of the three key IRGs on the malignant biological behavior of endometrial cancer. Thus, a characteristic model constructed using IRGs can predict the survival, chemotherapeutic drug sensitivity, and immunotherapy response in patients with endometrial cancer.

## INTRODUCTION

Endometrial cancer (EC) is one of the most common gynecological tumors in the United States, Europe, and other developed countries. Most patients with EC are diagnosed at an early stage and have enhanced survival with effective treatment. However, most patients with advanced EC are incurable [1, 2]. Therefore, identifying novel molecular players that function as diagnostic and prognostic biomarkers and potential therapeutic targets for EC is crucial.

The tumor microenvironment (TME), comprising cancer cells, non-tumor cells, and the extracellular matrix, plays significant roles in intra-tumoral crosstalk [3]. Cancer stem cells (CSCs), capable of self-renewal and differentiation [4], contribute to cancer recurrence, chemotherapy resistance, and tumor progression [5].

The inflammatory response is an important feature of tumors [6]. Both local and systemic inflammation contribute to tumor-related inflammation. Local inflammation promotes immune evasion and chemotherapeutic drug resistance by forming an inflammatory microenvironment, thereby promoting angiogenesis and metastasis to advance tumor progression [7]. The increased tumor mutational burden contributes to a chronic inflammatory response [8]. Our team previously developed the Naples score using blood inflammatory and nutritional indicators and demonstrated that this score could predict survival in patients with EC [9, 10]. However, only a few studies have investigated the role of inflammation in EC [11, 12]. Thus, the role of inflammatory response-related genes (IRGs) in EC prognosis remains unclear.

Herein, we hypothesized that IRGs could affect EC progression and patient prognosis. Thus, this study aimed to build an IRGs-based signature to predict patient prognosis and the effects of immunotherapy and chemotherapy. We also experimentally verified the effects of IRGs on the malignant biological behavior of EC cells to provide novel insights regarding the prognosis and efficacy of immunotherapy and precision medicine in EC.

## RESULTS

In this study (Supplementary Figure 1), an IRG-based gene signature was constructed to predict patient prognosis better than the existing models. This signature can also predict the curative effect of patients with EC to immunotherapy and commonly used chemotherapeutic drugs. Finally, we verified that the characteristic genes (LAMP3, MEP1A, and ROS1) in the IRGs could affect the malignant behavior of EC.

### Construction of a prognostic model

**Figure 1:**
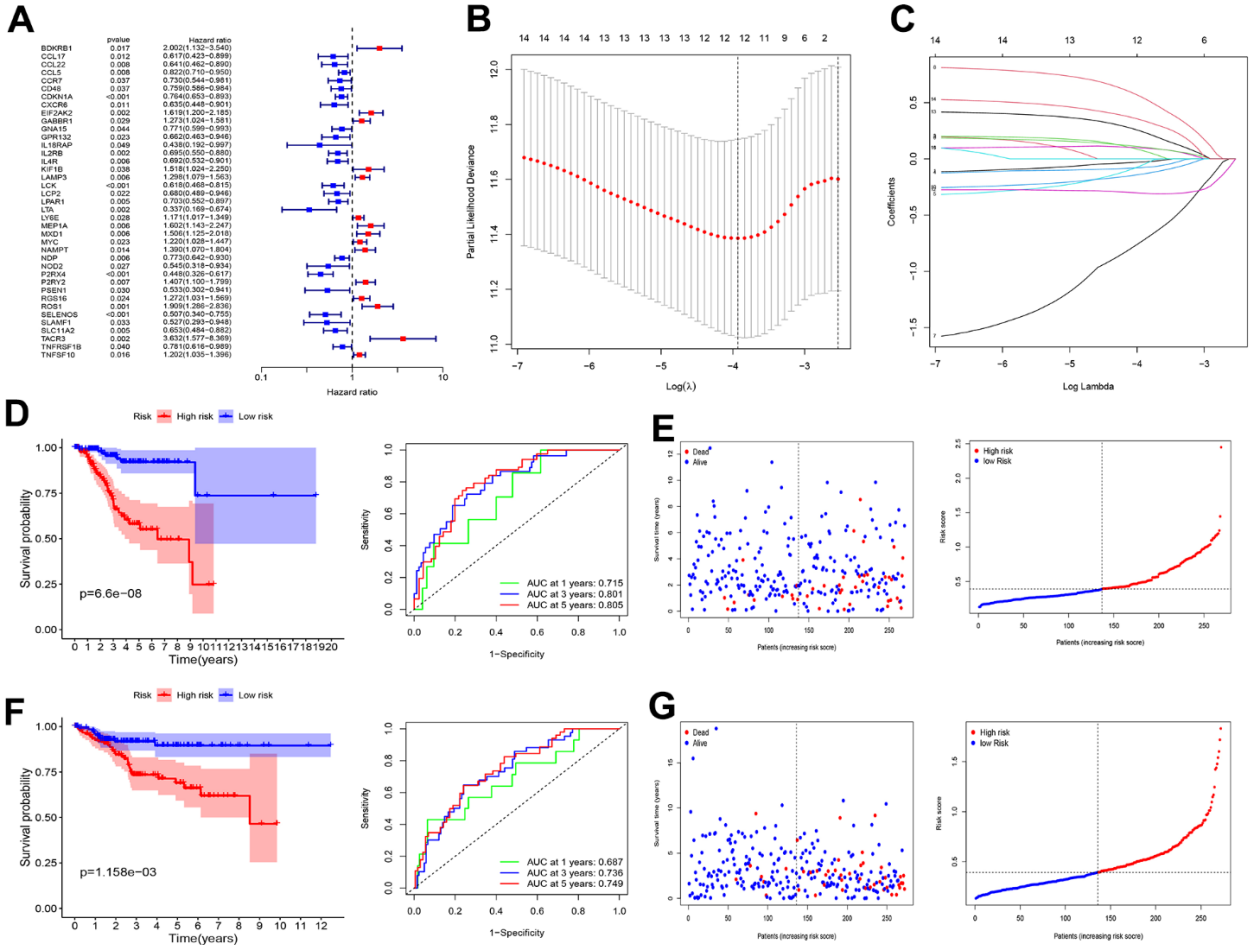
**Screening of prognosis RBP and construction of prognosis model.** (**A**) Univariate Cox regression analysis to identify the candidate prognosis-related hub IRGS in UCEC. (**B**) Partial likelihood deviation was plotted relative to the logarithm of lambda in 13-fold cross-validation. (**C**) The trajectory graph of each variable. (**D**) Survival curves and ROC curves of high and low risk groups in the training group. (**E**) The risk score value of each sample, the survival status ranked from low to high-risk scores in the training group. (**F**) Survival curves and ROC curves of high and low risk groups in the test group. (**G**) The risk score value of each sample, the survival status ranked from low to high-risk scores in the test group.

Univariate Cox regression analysis showed that 39 of the 200 IRGs were associated with overall survival (OS; OS was the duration from surgery to death expressed in months) (Figure 1A). We screened 39 prognostic IRGs using LASSO-Cox regression analysis (Figure 1B, 1C). Risk score = e^(0.1286 × Exp [GABBR1]) + (0.0212 × Exp [LAMP3]) + (−0.1382 × Exp [LCK]) + (−0.1231 × Exp [LPAR1]) + (0.0951 × Exp [MEP1A]) + (0.3928 × Exp [MXD1]) + (−0.0676 × Exp [NDP]) + (−0.2482 × Exp [P2RX4]) + (0.0128 × Exp [P2RY2]) + (−0.2786 × Exp [PSEN1]) + (0.1288 × Exp [ROS1]) + (−0.1594 × Exp [SLC11A2]) + (0.1905 × Exp [TNFSF10])^. Kaplan−Meier (KM) analysis of the training group showed differences in survival between the high- and low- risk groups (Figure 1D). In the training group, the areas under the receiver operating characteristic curves (AUCs) for 1-, 3-, and 5-year OS were 0.715, 0.801, and 0.805, respectively (Figure 1D). Patient survival status and risk score distribution in the training group are shown in Figure 1E. This model exhibits a certain predictive ability. The same formula was used for the test group. This risk score also showed a good predictive ability in the test group (Figure 1F, 1G).

### Establishment of the nomogram

**Figure 2:**
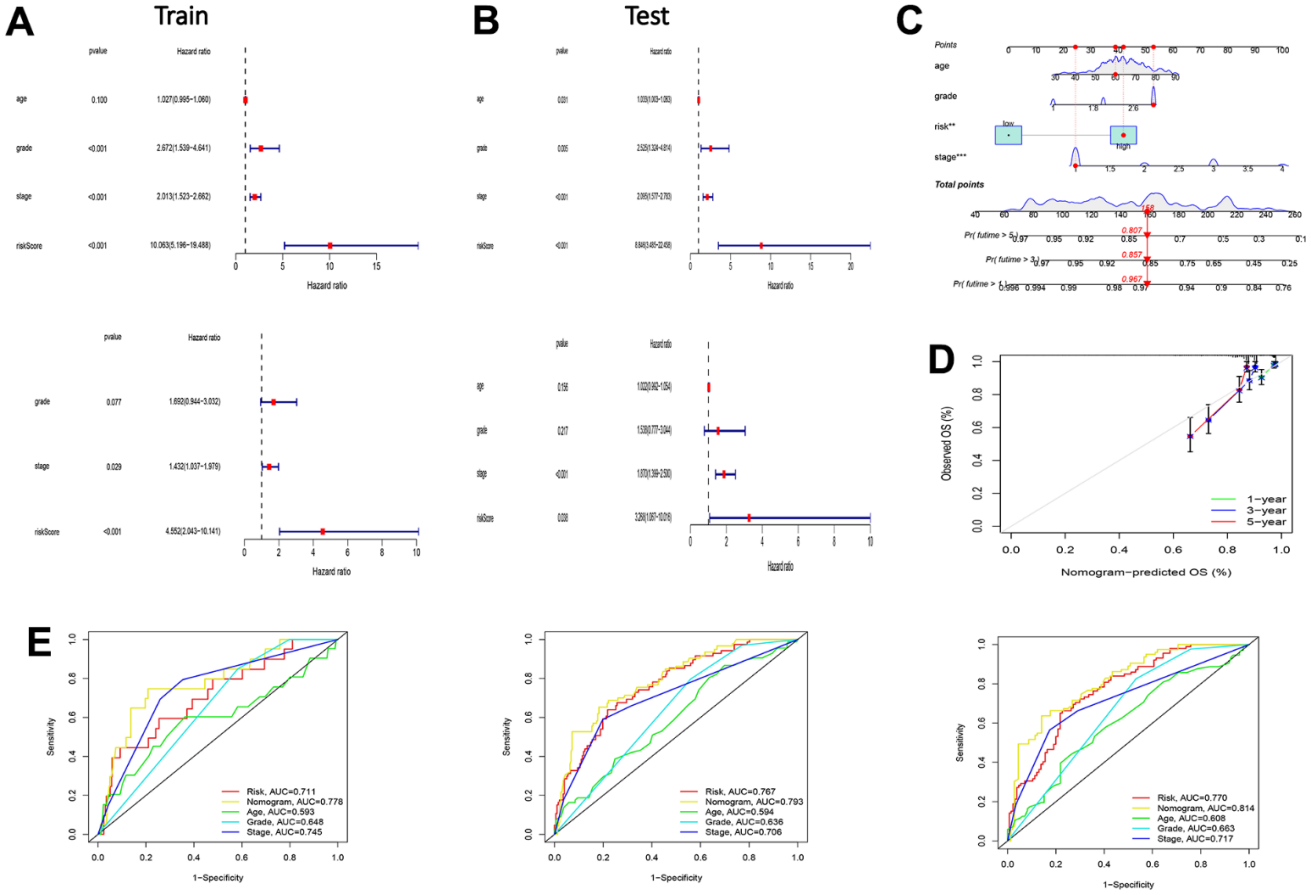
**Test of risk prediction model for UCEC patients and nomogram quantitative prediction of survival time and correction of UCEC patients.** (**A**) Univariate and multivariate analysis were performed to assess the clinicopathological prognostic value of the prediction model in the training group. (**B**) Univariate and multivariate analysis were performed to assess the clinicopathological prognostic value of the prediction model in the test group. (**C**) Nomogram for predicting the 1-, 3-, and 5-year OS of UCEC patients. (**D**) Calibration curves for the prediction of 1-, 3- or 5-year overall survival of UCEC patients. (**E**) ROC curves for predicting the 1-, 3-, and 5-year OS of UCEC patients.

The risk score was an independent prognostic factor (Figure 2A, 2B). We then built a nomogram to quantitatively predict the prognosis patients with EC (Figure 2C). Calibration curves showed high accuracy and validity (Figure 2D). The AUCs for 1-, 3-, and 5-year OS were 0.778, 0.793, and 0.814, respectively (Figure 2E).

### Comparison between models

**Figure 3:**
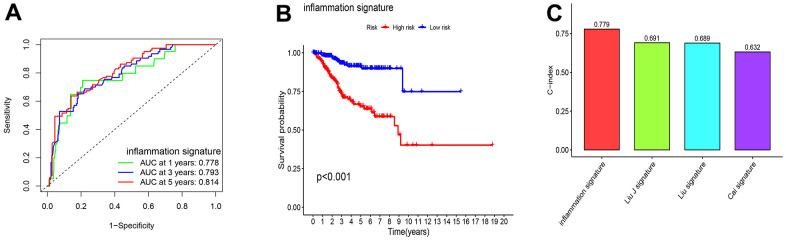
**The comparison between model for IRGs and the existing model for signatures.** (**A**, **B**) Survival curves and ROC curves of high and low risk groups in the model constructed by us. (**C**) C-index comparison of inflammatory models with other models.

To verify the effectiveness of the constructed model, we also compared it with existing models. Figure 3A, 3B displays the models constructed by our group. The C-index results showed that the model constructed using the IRGs was superior to the models constructed by Cai, Liu, and Liu J [13–15] (Figure 3C).

### Association analysis between functional characteristics and risk score

**Figure 4:**
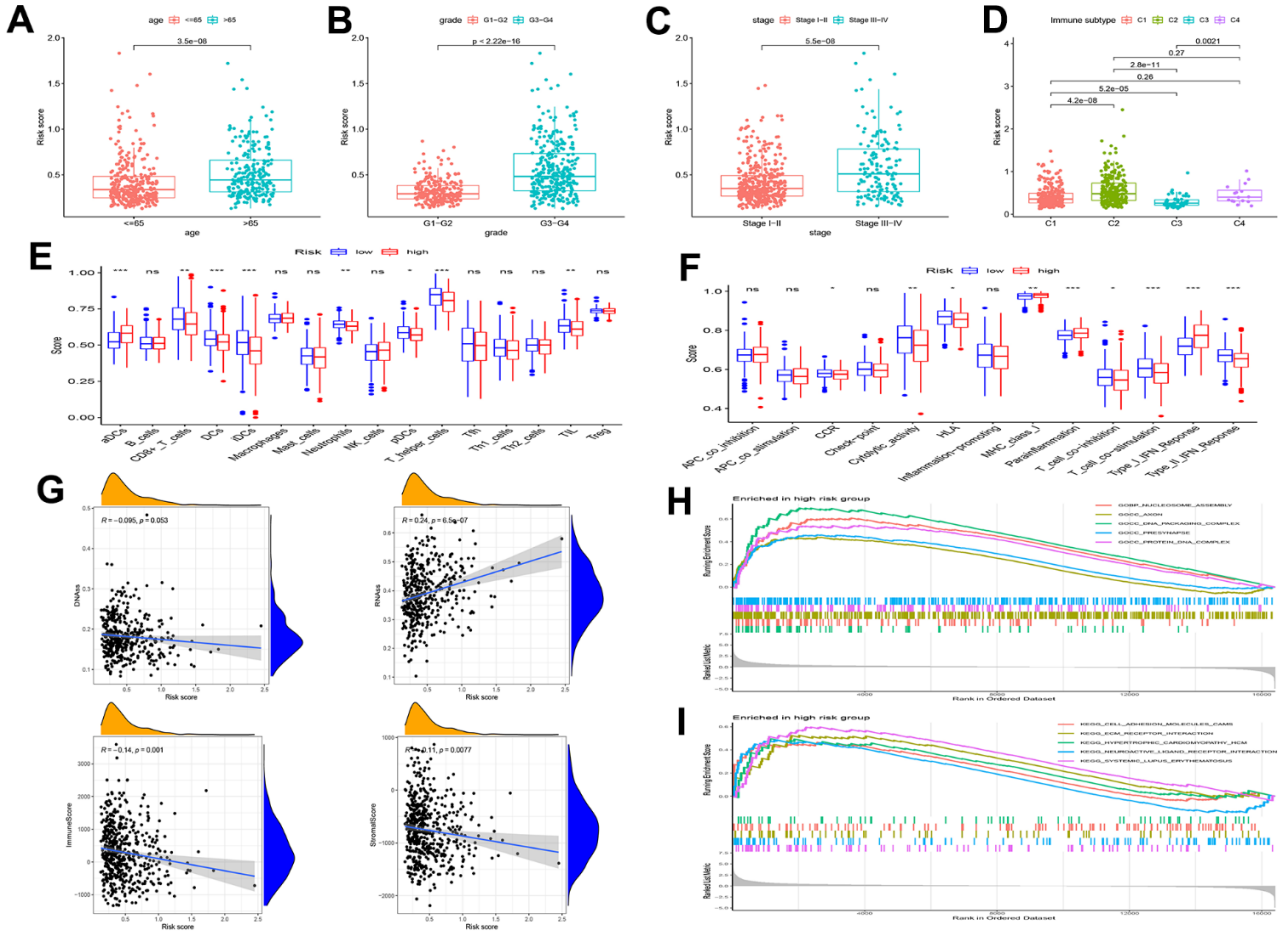
**Gene set enrichment analysis (GSEA) of biological functions and the association between risk score and tumor microenvironment.** The risk score in different groups divided by age (**A**), grade (**B**), stage (**C**) and immune subtype (**D**). Comparison of the risk score in different immune infiltration. (**E**, **F**) The relationship between risk score and the scores of 16 immune cells and 13 immune-related functions were showed in boxplots. (**G**) The relationship between risk score and DNAss, RNAss, Stromal Score and Immune Score. (**H**, **I**) GSEA showed eleven pathways enriched in the high-risk group. P values were showed as: ns, not significant; *P < 0.05; **P < 0.01; ***P < 0.001.

Association analysis between risk score and clinical characteristics showed that the risk score was higher in the >65 age groups (P < 0.001), tumor stage III–IV (P < 0.001), or tumor grade 3–4 (P < 0.001) than in the ≤65 age groups, tumor stage I–II, or tumor grade 1–2 (Figure 4A–4C). Moreover, this risk score was higher in the immune subtypes (C2) with a poorer prognosis (Figure 4D).

Single-sample Gene Set Enrichment Analysis (ssGSEA) revealed that the fractions of CD8^+^T cells, DCs, iDCs, pDCs, T helper cells, and TIL were significantly decreased in the high-risk group (Figure 4E). Moreover, CCR, type II IFN response score, and T cell co-stimulation were significantly decreased in the high-risk group (Figure 4F).

RNA stemness scores (RNAss) and DNA stemness scores (DNAss) were used to evaluate CSCs [16]. The results revealed that the risk score significantly positively correlated with RNAss, but negatively correlated with immune and stromal scores (Figure 4G). In addition, we assessed the relationship between pathway enrichment and risk scores. GO and KEGG enrichment analyses showed that nucleosome assembly, DNA packaging complex, presynapse, protein-DNA complex, ECM receptor interaction, and cell adhesion molecules were significantly enriched in the high-risk group (Figure 4H, 4I).

### Predicting the effects of anti-cancer treatment and immunotherapy sensitivity

**Figure 5:**
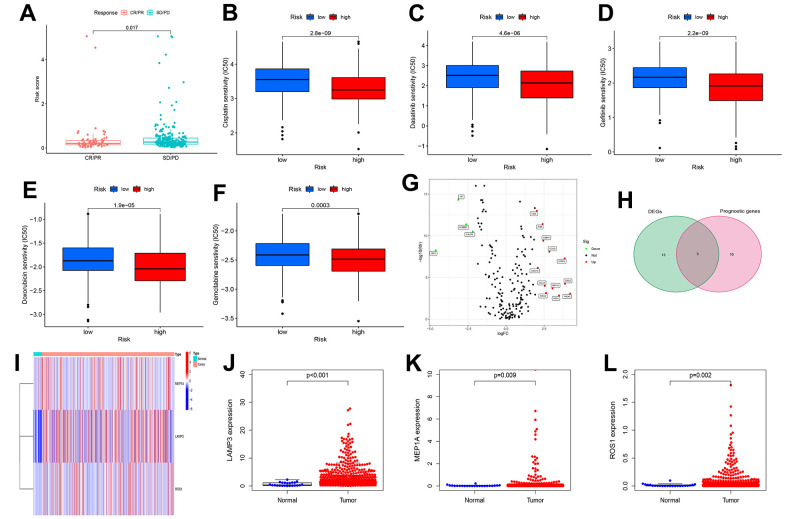
**Risk scores are associated with immunotherapy and chemotherapeutic drug response and identification of differentially expressed genes.** (**A**) The correlation between risk scores and immunotherapy response. (**B**–**F**) The correlation between risk scores and Chemotherapeutic drug sensitivity. (**G**) volcano plot of Differentially expressed IRGs (DEIRGs): upregulated DEIRGs are indicated by red dots, and downregulated DEIRGs are indicated by green dots. (**H**) The DEIRGs were intersected with the genes included in the model. (**I**) Heat map of LAMP3, MEP1A and ROS1 between tumor and normal tissues. Box plots showed the expression of LAMP3 (**J**), MEP1A (**K**) and ROS1 (**L**) in normal and UCEC tissues from TCGA.

To further verify the relationship between risk score and immunotherapy sensitivity, we downloaded the dataset from the IMvigor210 database. Figure 5A shows that the risk scores were significantly higher in patients with progressive diseases (PD) or stable disease (SD) than in those with complete response (CR) or partial response (PR). Figure 5B–5F showed that the high-risk group was more sensitive to the chemotherapeutic drugs cisplatin, dasatinib, doxorubicin, gefitinib, and gemcitabine than the low-risk group. Therefore, those patients with EC in the low-scoring group were more likely to benefit from immunotherapy. Although patients in the high-risk group have a poor survival prognosis, we can use more sensitive chemotherapy drugs in the high-risk group to improve their prognosis.

### Identification of differential genes

We identified 16 differentially expressed IRGs (DEIRGs) (Figure 5G). Figure 5H, 5I shows that the intersection of the DEIRGs and 13 IRGs (in the prognostic model) yielded three common genes (MEP1A, LAMP3, and ROS1). The expression of MEP1A, LAMP3, and ROS1 was upregulated in the uterine corpus EC (UCEC) tissue-based TCGA datasets (Figure 5J, 5K). The qRT-PCR results showed that MEP1A, LAMP3, and ROS1 (Supplementary Figure 2A–2C) were highly expressed in human EC tissues, which is consistent with our analysis of the TCGA-UCEC dataset. Analysis of clinicopathological parameters of EC patients showed that the expression of the three genes (LAMP3: Supplementary Figure 2D–2F, MEP1A: Supplementary Figure 2G–2I, and ROS1: Supplementary Figure 2J–2L) were higher in the >65 age (P > 0.05), tumor stage III–IV (P < 0.05), or lymph node (LN) metastasis (P < 0.05) groups than in the ≤65 age groups, tumor stage I–II, or no LN metastasis groups.

### Cell functional experimental validation of three key genes

**Figure 6:**
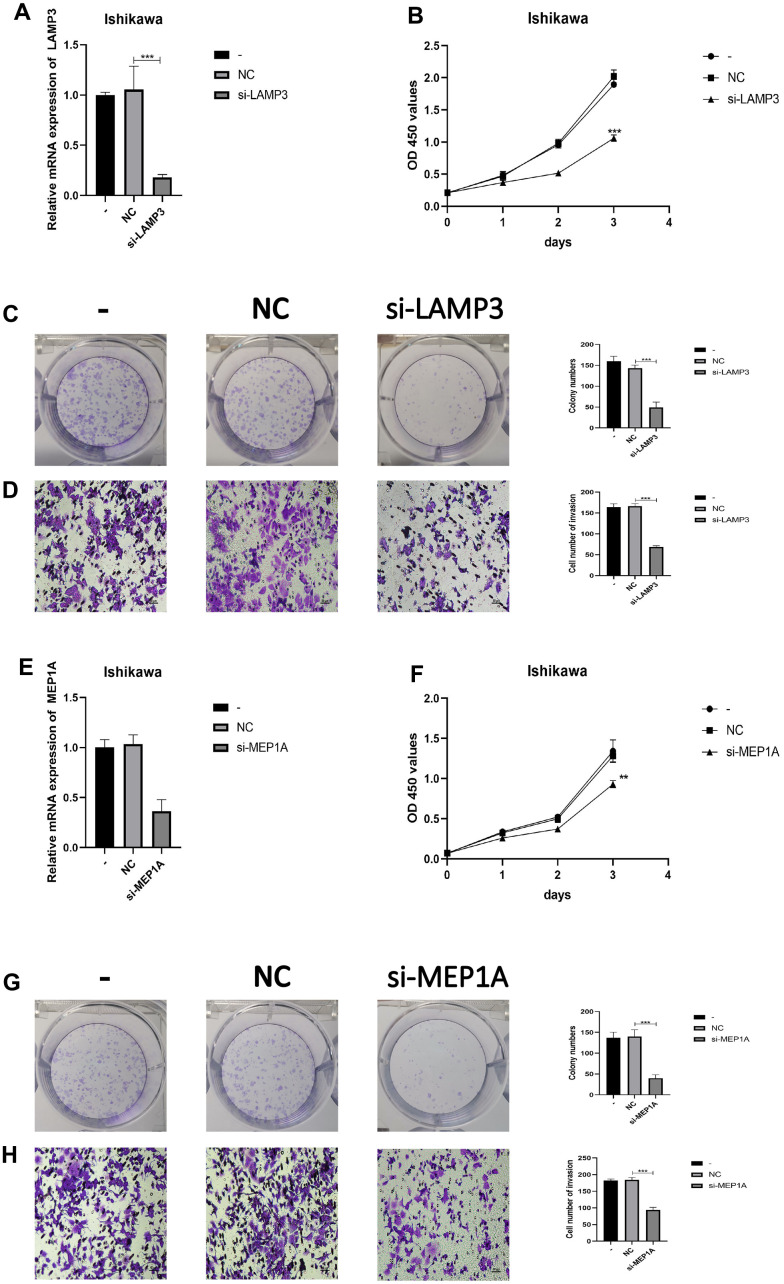
**LAMP3 and MEP1A regulates the biological behavior of Ishikawa cell lines.** (**A**) PCR was used to verify knockdown efficiency of LAMP3. (**B**, **C**) CCK-8 and colony formation assays were used to evaluate the proliferation effect of LAMP3. (**D**) Effect of LAMP3 on invasion assessed using the Transwell assay. (**E**) PCR was used to verify knockdown efficiency of MEP1A. (**F**, **G**) CCK-8 and colony formation assays were used to evaluate the proliferation effect of MEP1A. (**H**) Effect of MEP1A on invasion assessed using the Transwell assay.

**Figure 7:**
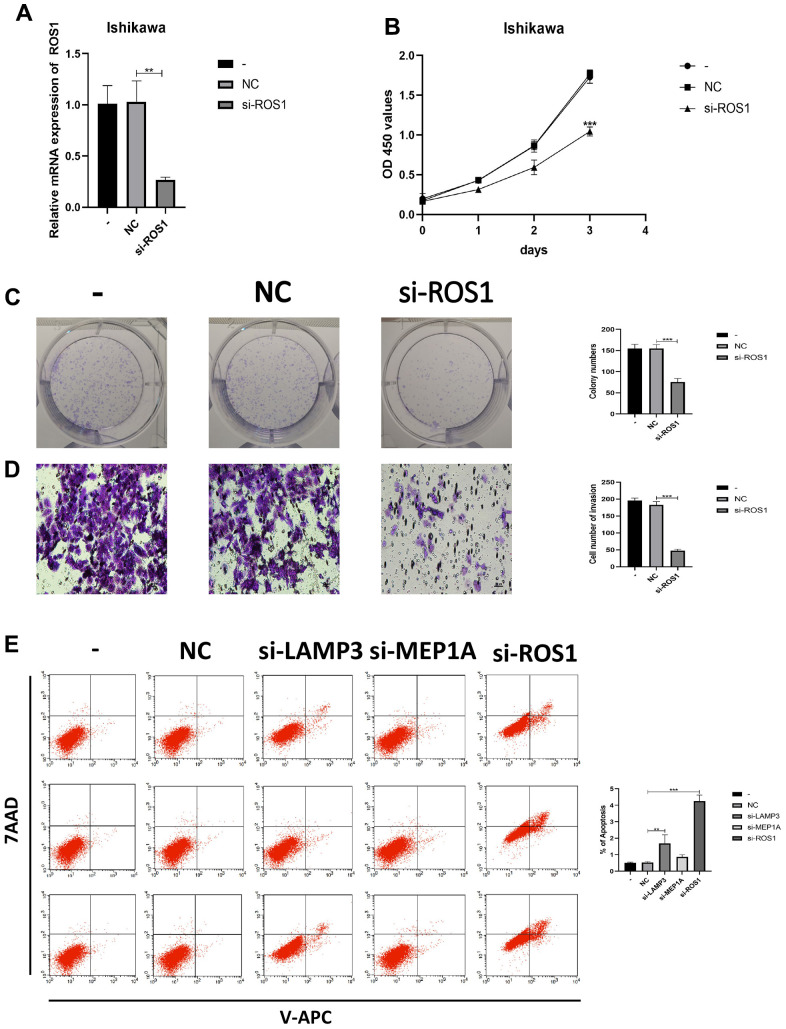
**IRGs regulates the biological behavior of Ishikawa cell lines.** (**A**) PCR was used to verify knockdown efficiency of ROS1. (**B**, **C**) CCK-8 and colony formation assays were used to evaluate the proliferation effect of ROS1. (**D**) Effect of ROS1 on invasion assessed using the Transwell assay. (**E**) Cell apoptosis assay was used to determine the effect of LAMP3, MEP1A and ROS1.

To further verify the role of LAMP3, MEP1A, and ROS1 in EC, we knocked down the expression of LAMP3 (Figure 6A), MEP1A (Figure 6E), and ROS1 (Figure 7A) in EC cells (Ishikawa cells) and examined their siRNA effects on the cells using PCR. Silencing of these three genes inhibited proliferation (LAMP3: Figure 6B, 6C, MEP1A: Figure 6F, 6G, and ROS1: Figure 7B, 7C) and invasion (LAMP3: Figure 6D, MEP1A: Figure 6H, and ROS1: Figure 7D) of ECs. In Ishikawa cells, LAMP3 and ROS1 knockdown promoted apoptosis, whereas MEP1A knockdown did not (Figure 7E). These results suggested that LAMP3, MEP1A, and ROS1 function as oncogenes in EC.

## DISCUSSION

EC is one of the most common tumors affecting the female reproductive system. Currently, EC treatment mainly employs surgical intervention alone in the early stages and combines surgery with other adjuvant treatments in advanced stages [17]. However, last-stage EC is highly invasive and migratory, and the efficacy of treatments is low [18]. If EC is not effectively diagnosed and treated in its early stages, the five-year survival rate for advanced endometrial cancer drops to 16–45% compared with 95% in its early stages [1, 2]. Therefore, it is important to explore novel prognostic biomarkers and druggable targets for EC.

The main risk factors for EC are exposure to endogenous and exogenous estrogen, diabetes, obesity, late-onset menopause, nulliparity, and older age [19]. However, recent research has suggested that inflammation is an important risk factor for EC [20–23]. Interestingly, in our independent retrospective cohort study, we reached similar conclusions, suggesting that inflammatory markers in the blood are strongly associated with EC prognosis [10, 24]. Although multiple clinical cohorts and retrospective studies have revealed that inflammation is a risk factor for EC, there are few studies on the underlying mechanisms of the inflammatory response in the occurrence and development of EC. Using second-generation sequencing technology, it is now possible to reveal the association between the inflammatory response and EC at the gene level. Precision medicine requires new and improved models to predict the survival and efficacy of immunotherapy and chemotherapy drugs in patients with EC.

The TCGA-UCEC dataset was downloaded for this study. Univariate analysis of TCGA-UCEC dataset identified 39 prognostic IRGs, and LASSO regression was used to build a new gene signature containing 13 IRGs. We then constructed a nomogram based on the clinical information of UCEC patients (age, stage, and grade) and compared this signature with the models constructed by Cai [13], Liu [14], and Liu J [15]. The results showed that our model is superior to the existing models. The survival of EC patients is dependent on predictive factors such as age, stage, and grade [25, 26]. Therefore, our final nomogram, based on these clinical data and risk scores, had a better predictive power. Furthermore, we analyzed the risk score and clinical information of the patients, and the results revealed that patients with clinical risk factors (> 65, grade 3–4, stage III–IV) also had higher risk scores, proving that risk scores were consistent with clinical risk factors. A study based on TCGA database categorized tumors into six immune infiltration subtypes (C1–C6) [27]. Immune subtypes were closely related to progression-free survival (PFS) and OS; C1 and C2 had poor prognoses, C3 had the best survival, and C4 and C6 had the worst prognoses. In this study, high-risk scores with worse prognoses were associated with immunotypes C1, C2, and C4.

Inflammation and the inflammatory TME drive tumor growth, metastasis, progression, and initiation [28]. However, there have been few studies on inflammation in EC, and the mechanisms of IRGs in EC remains unclear. The effects of the risk scores and IRGs included in the model on the prognostic mechanism of patients with EC have not been clarified. Therefore, we further explored this model. Results of ssGSEA showed that the fractions of TIL, T helper cells, pDCs, DCs, iDCs, and CD8^+^T cells, and were significantly decreased in the high-risk group. This suggests that our model is sensitive to changes in immune function. Previous studies have shown that changes in these immune-related cells affect the prognosis of cancer patients [29, 30]. CSCs are resistant to therapeutic drugs, and their presence may affect patient outcomes [31–33]. Our results showed that the risk score was negatively correlated with the immune and stromal scores and significantly positively correlated with RNAss. This suggests that the risk score may predict EC patient prognosis by analyzing their stemness characteristics. Previous studies have shown that T and B cells affect tumor progression in EC by activating the IFN and TFN inflammatory pathways via the IgA pathway [34, 35]. These findings suggest that immune cells in the TME may function by influencing inflammatory pathways in EC tumor cells.

Currently, clinical treatments exist for only some types of endometrial carcinomas [36, 37]; however, only a few studies have evaluated the sensitivity of patients with EC to chemotherapeutic agents and immunotherapy [38]. Interestingly, in this study, patients in the high-risk group had a lower response rate to immunotherapy, but more sensitive to chemotherapy drugs (cisplatin, dasatinib, doxorubicin, gefitinib, and gemcitabine) than the low-risk group. To date, cyclophosphamide, doxorubicin, and cisplatin (CAP) combination chemotherapy is used as a first-line treatment for treating patients with EC [19, 39–42]. Therefore, in our model, conventional CAP chemotherapy regiments are recommended over expensive immunotherapy for high-risk groups. These data can guide clinical chemotherapeutic drug application and predict drug sensitivity in advance. Although immunotherapy is not the preferred treatment for patients with EC, it has shown great therapeutic potential in Food and Drug Administration-approved phase I immunotherapy trials [43]. The high-risk group in our model was more likely to be unresponsive to immunotherapy, which may be related to the disturbance of inflammatory pathways downstream of the immunotherapy targets. In summary, the risk scores constructed in this study show great potential for predicting immunotherapy responses and chemotherapeutic drug sensitivity. This suggests that the risk scores can be used to predict the efficacy of chemotherapy drugs and immunotherapy in patients with EC, thereby contributing to the development of precision therapy.

However, this study has some limitations. There was no external validation due to the lack of other datasets with UCEC clinical data. In addition, the effectiveness of the model in clinical practice is unknown, and we intend to conduct further research in the future. Nonetheless, our exploration of IRGs also provides a reference for studying inflammatory responses in UCEC. Thus, these findings might have potential clinical application for better prognostic management of patients with EC. Additionally, the genes in this model may serve as molecular targets for EC therapy.

## CONCLUSIONS

The IRGs-based model that we constructed for the first time have stable predictive power for patient prognosis. This model can be used as a potential prognostic index for patients with EC. Meanwhile, we also provide a theoretical basis for the future studies on inflammatory response in EC. Overall, this study could help reveal the role of IRGs in TME, immunotherapy response, and chemotherapeutic drug resistance, which is crucial for personalized tumor treatment and precision medicine. These findings might have potential clinical application for better prognostic management of patients with EC.

## MATERIALS AND METHODS

### Data acquisition

The clinical information and RNA sequencing datasets (FPKM) of patients with UCEC were downloaded from TCGA (https://portal.gdc.cancer.gov/) database. We obtained an RNA sequencing dataset of 552 UCEC and 35 normal tissues (23 tumors had matching adjacent normal tissues). After excluding patients with EC with incomplete clinical information or who were lost to follow-up, 541 patients with EC were enrolled. The IRGs were selected from the Molecular Signatures database (HALLMARK_INFLAMMATORY_RESPONSE (gsea-msigdb.org)). Immunotherapy data were obtained from IMvigor210.

### Construction of model and nomogram

We used univariate Cox regression analysis to analyze the 200 IRGs from the Molecular Signatures database to identify prognostic IRGs associated with OS. Because only TCGA contained prognostic information for patients with EC, we could only test our model internally. The TCGA-UCEC patients were randomly and equally divided into training (n = 272) and test (n = 269) groups. We then used LASSO-penalized Cox regression analysis on the training set [44]. Risk score was calculated as e^sum(expression of each gene × corresponding coefficient)^. The UCEC patient prognostic data in the training group were divided into high- and low-risk subgroups based on the median risk score as the cutoff value. The test group was used to verify the validity of the proposed model. R packages “regplot” and “rms” were used to build a nomogram by combining the clinical information of patients with EC and compared with previously established models. GSEA was used to analyze KEGG and GO enrichment in this model. Correlation analysis of immune function was based on ssGSEA [45].

### Tumor microenvironment analysis

The R (version 4.1.1) package “ESTIMATE” was used to analyze infiltration of immune and stromal cells [46]. We extracted Cancer stem cells (CSCs) data for each patient from their epigenomic and transcriptomic data to measure CSCs characteristics. Spearman analysis was used for statistical analysis.

### Predicting the chemotherapeutic immunotherapy response

The packages in R were used to evaluate the sensitivity of patients to chemotherapy drugs. Half-maximal inhibitory concentration (IC50) was calculated to compare drug sensitivity. Wilcoxon rank test and the “ggplot” package was used to visualize the result. Data sets from IMvigor210 were used to evaluate the efficacy of immunotherapy in cancer patients.

### Screening of key genes in the model

We utilized the “limma” package in R software to identify the differentially expressed IRGs (DEIRGs) among the 200 selected genes. Our selection criteria for DEIRGs were |log2 fold change (FC)| ≥ 2 and false discovery rate < 0.05. Subsequently, we generated heatmaps and volcano plots using the “pheatmap” package. We then overlapped the DEIRGs with the genes in the model to identify the key genes. These key genes not only play a significant role in the model but may also contribute to tumor development, and further experiments will be conducted to verify their importance.

### Human tissue specimens

We obtained 43 samples of normal endometrial tissues and 37 samples of uterine corpus EC tissues from Shengjing Hospital of China Medical University, China, between 2019 to 2021. Complete clinicopathological information was obtained for 32 patients with EC. All patients provided informed consent. The pathologic type of all cases of EC was endometrial adenocarcinoma, and the pathological diagnosis was made by two experienced pathologists in accordance with the International Federation of Gynecology and Obstetrics (FIGO 2009). None of the patients received any form of hormone, radiotherapy, chemotherapy, or treatment prior to surgery.

### Transfection of cells

SiRNA sequences targeting LAMP3, MEP1A and ROS1, along with their respective negative control (NC) counterparts, were procured from GenePharma (Shanghai, China). Lipofectamine 3000 (Invitrogen) was used to transfect cells with the siRNA according to the manufacturer’s instructions for subsequent experiments. Sequences of siRNA are listed in Supplementary Table 1.

### qRT-PCR

Total RNA was extracted using TRIzol reagent (Vazyme, Nanjing, China). Subsequently, cDNAs were synthesized using Prime Script RT-polymerase (Vazyme). SYBR Green Premix (Vazyme) with specific PCR primers (Sangon Biotech, Shanghai, China) were used to detect the expression level of corresponding gene RNA. The primer sequences are provided in Supplementary Table 2. The fold-changes were calculated using the 2^−ΔΔCT^ method.

### Cell culture

The Ishikawa cells were cultured in RPMI 1640 medium (Gibco, Carlsbad, CA, USA), supplemented with 10% fetal bovine serum (FBS) (Gibco) and 1% penicillin–streptomycin. The cells were maintained in a humidified incubator at 37° C with 5% CO2.

### Colony formation assay

To investigate the impact of LAMP3, MEP1A and ROS1 expression on cell proliferation, Ishikawa cells were transfected with NC-siRNA or siRNA targeting the genes of LAMP3, MEP1A, and ROS1. Additionally, a blank group without siRNA was included. Following transfection, 1000 cells were seeded to each well of 6-well culture plates and incubated for two weeks. Afterward, the cells were stained with 0.1% crystal violet, and the number of colonies was counted using light microscopy.

### CCK-8 assay

Ishikawa cells were seeded in 96-well plates, and to each well, CCK-8 reagent (10 μL) (Dojindo, Kumamoto Japan) was added. The cells were then incubated at 37° C with 5% CO2 for 3 h. The OD 450 nm was measured using a microplate reader at 0, 24, 48, and 72 hours after treatment to determine the cell viability.

### Cell invasion assay

Transwell chambers (Corning, NY, USA) (pore size:8μm) were utilized to evaluate cell invasion ability. Prior to seeding cells, the chambers were coated with Matrigel solution (BD, Franklin Lakes, NJ, USA), and the upper chamber was filled with 200μl serum-free medium. The lower chamber was supplemented with 10%FBS serum. After incubation for 24 hours, the invading cells that penetrated the Matrigel and reached the lower surface of the membrane were fixed with 4% paraformaldehyde and stained with 0.1% crystal violet. Images were obtained using a fluorescence inversion microscope (200× magnification) and subsequently analyzed.

### Apoptosis assay

After cell transfection, 10^6^ cells from each group were washed and stained Annexin V-APC and 7AAD (Elabscience, Wu Han, China) at room temperature for 15 min. Flow cytometry (BD FACSCalibur, New Jersey, USA) was used to evaluate the proportion of apoptotic cells (low right corner of the flow cytometry graph regarded as apoptotic cells).

### Statistical analysis

The GraphPad Prism 8 (GraphPad Software, CA, United States) were used for statistical analysis. Each experiment was repeated three times independently. Data are presented as mean ± standard deviation (SD). Two-way comparisons between groups were analyzed using the t-test, and multiple group comparisons were analyzed using one-way ANOVA. Statistical significance was set at p < 0.05.

### Availability of data and materials

All data generated or analyzed during this study are included in this article.

### Consent for publication

We have obtained consent to publish this paper from all the participants of this study.

## Supplementary Material

Supplementary Figures

Supplementary Tables
