# Supplementary material for: A novel predictive model based on inflammatory response-related genes for predicting endometrial cancer prognosis and its experimental validation
Source: Aging (Albany NY). 2023 Jun 5;15(11):4844–60. doi: 10.18632/aging.204767 (PMC10292875; doi:10.18632/aging.204767)
Supplement: Supplementary Figures [file aging-15-204767-s001.pdf]

SUPPLEMENTARY FIGURES

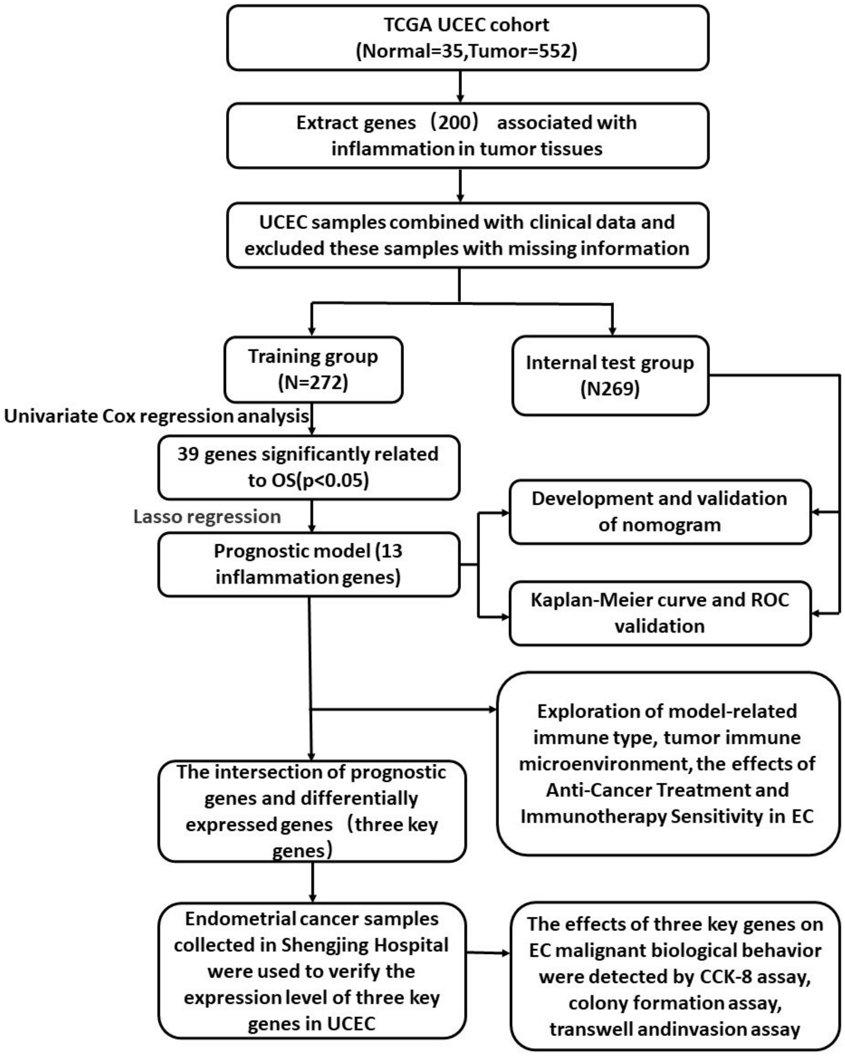

Supplementary Figure 1. Comprehensive prognostic value analysis framework of inflammatory response-related genes (IRGs) in uterine corpus endometrial carcinoma (UCEC) patients based on TCGA database.

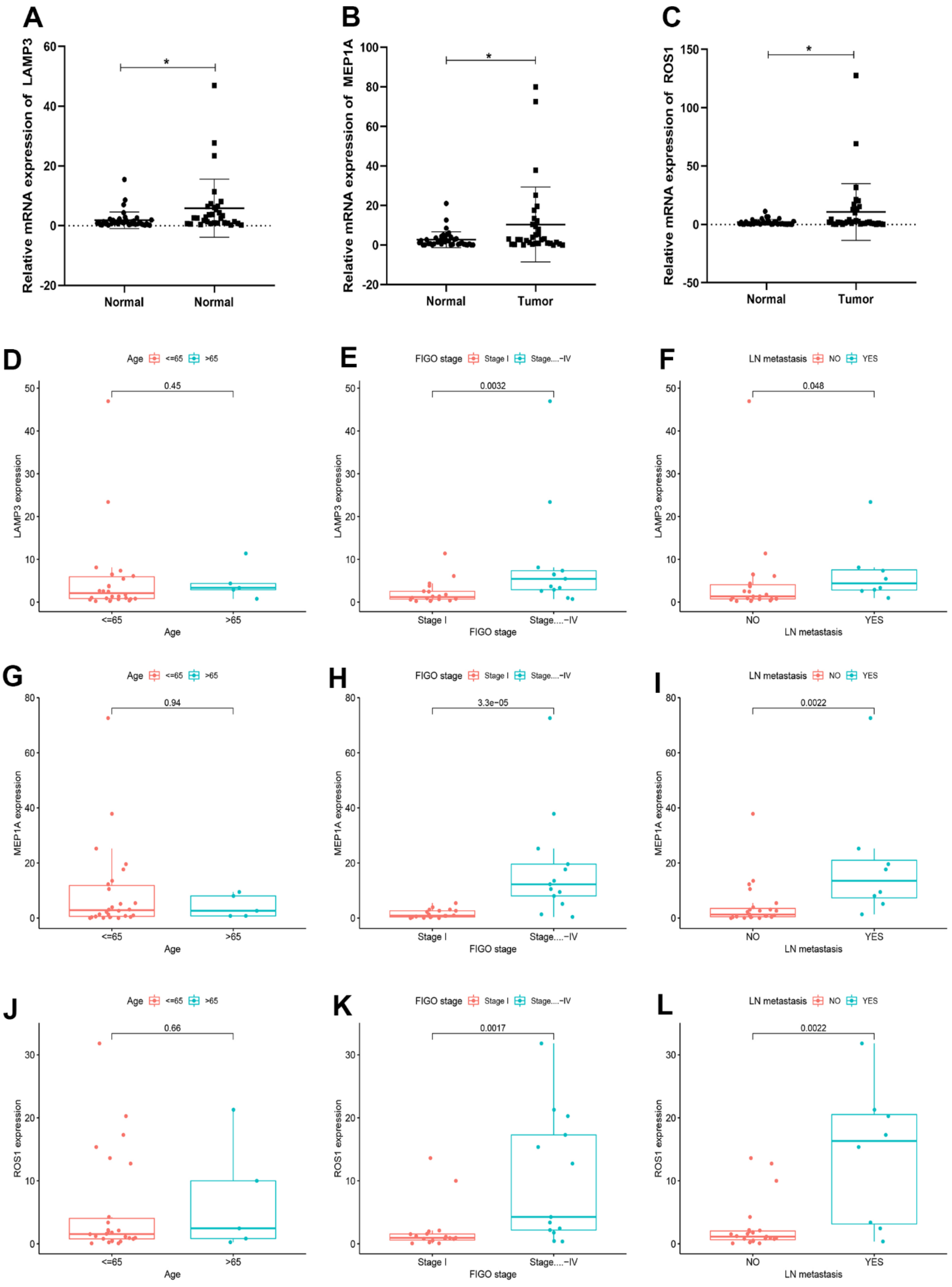

Supplementary Figure 2. Relationships between three key IRGs expression in EC and clinicopathological parameters. The results of qRT-PCR showed the expression of LAMP3 (**A**), MEP1A (**B**) and ROS1 (**C**) in normal endometrial tissue (n=43) and human endometrial carcinoma tissue (n=37). The expression of LAMP3 in different groups divided by age (**D**), stage (**E**) and LN-metastasis (**F**). The expression of MEP1A in different groups divided by age (**G**), stage (**H**) and LN-metastasis (**I**). The expression of MEP1A in different groups divided by age (**J**), stage (**K**) and LN-metastasis (**L**).
