# Supplementary material for: A novel predictive model based on inflammatory response-related genes for predicting endometrial cancer prognosis and its experimental validation
Source: Aging (Albany NY). 2023 Jun 5;15(11):4844–60. doi: 10.18632/aging.204767 (PMC10292875; doi:10.18632/aging.204767)
Supplement: Supplementary Tables [file aging-15-204767-s002.pdf]

SUPPLEMENTARY TABLES

Supplementary Table 1. Primer sequence.

Gene name	Primer sequence
LAMP3	Forward: GAACAGAGCCTCCAGTTGTCAGC
	Reverse: TCAGACGAGCACTCATCCACATTTC
MEP1A	Forward: TTTACCCAAAGAGGAAGCAGCAGTG
	Reverse: CCTTCACCAACTTGCGAACATTGC
ROS1	Forward: AGCTGTGCGTATTGTGGAGAGTTG
	Reverse: TGCGAGGTAGGATGAGATGGGAAG
GAPDH	Forward: CAGGAGGCATTGCTGATGAT
	Reverse: GAAGGCTGGGGCTCATTT

Supplementary Table 2. Sequence of siRNA.

Name	Sequence
LAMP3-Homo-280	Sense (5'-3'): CACGAUGGCAGUCAAAUGATT
	Antisense (5'-3'): UCAUUUGACUGCCAUCGUGTT
MEP1A-HOMO-606	Sense (5'-3'): GCACAACUUUGACACCUAUTT
	Antisense (5'-3'): AUAGGUGUCAAAGUUGUGCTT
ROS1-Homo-499	Sense (5'-3'): CCUACCAACUGCUCCCUUUTT
	Antisense (5'-3'): AAAGGGAGCAGUUGGUAGGTT
